# Fatty Acid Metabolism, Bone Marrow Adipocytes, and AML

**DOI:** 10.3389/fonc.2020.00155

**Published:** 2020-02-18

**Authors:** Yoko Tabe, Marina Konopleva, Michael Andreeff

**Affiliations:** ^1^Department of Laboratory Medicine, Juntendo University, Tokyo, Japan; ^2^Section of Molecular Hematology and Therapy, Department of Leukemia, The University of Texas MD Anderson Cancer Center, Houston, TX, United States; ^3^Section of Leukemia Biology Research, Department of Leukemia, The University of Texas MD Anderson Cancer Center, Houston, TX, United States

**Keywords:** fatty acid metabolism, fatty acid oxidation, bone marrow microenvironment, adipocyte, therapy resistance

## Abstract

Acute myeloid leukemia (AML) cells modulate their metabolic state continuously as a result of bone marrow (BM) microenvironment stimuli and/or nutrient availability. Adipocytes are prevalent in the BM stroma and increase in number with age. AML in elderly patients induces remodeling and lipolysis of BM adipocytes, which may promote AML cell survival through metabolic activation of fatty acid oxidation (FAO). FAO reactions generate acetyl-CoA from fatty acids under aerobic conditions and, under certain conditions, it can cause uncoupling of mitochondrial oxidative phosphorylation. Recent experimental evidence indicates that FAO is associated with quiescence and drug-resistance in leukemia stem cells. In this review, we highlight recent progress in our understanding of fatty acid metabolism in AML cells in the adipocyte-rich BM microenvironment, and discuss the therapeutic potential of combinatorial regimens with various FAO inhibitors, which target metabolic vulnerabilities of BM-resident, chemoresistant leukemia cells.

## Acute Myeloid Leukemia, the Bone Marrow (BM) Microenvironment, and Metabolism

**Figure 1:**
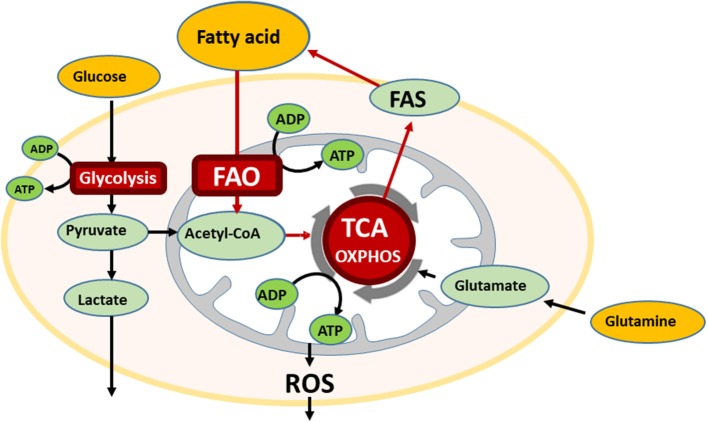
The bone marrow microenvironment reprograms energy metabolism in AML. AML cells utilize multiple metabolic pathways for energy production, glycolysis, oxidative phosphorylation (OXPHOS), and if fatty acid is available, also undergo fatty acid oxidation (FAO). In the oxidative stressed bone marrow (BM) microenvironment, AML cells are supplied free fatty acids by abundant BM adipocytes, and utilize for FAO. FAO involves a series of reactions to generate acetyl-CoA from fatty acid under aerobic conditions. Acetyl-CoA enters the TCA cycle.

In the bone marrow (BM) microenvironment, AML cells modulate their metabolic state to respond to extracellular stimuli, like hypoxia and nutrient availability, and can therefore undergo cell kinetic quiescence, proliferation, or differentiation (1). Glucose is metabolized to pyruvate through glycolysis during normal and pathological conditions, and, in the presence of oxygen, pyruvate can be further metabolized to acetyl-CoA that is oxidized in the Krebs cycle to drive oxidative phosphorylation (OXPHOS) and ATP generation. This process can generate 36 moles of ATP per mole of glucose that is 18 times more than that generated by glycolysis alone. Interestingly, OXPHOS is reportedly highly active in AML cells (2). In addition to glucose, proteins and fatty acids can also be metabolized to acetyl-CoA to drive the Krebs cycle and OXPHOS in ATP production (3) (Figure 1).

Acute myeloid leukemia (AML) is primarily a disease of the elderly, with 75% of AML patients greater being over 60 years old at diagnosis (4). In adult BM, adipocytes are the dominant stromal cell, which increase in number with advancing age. For example, it is estimated that adipocytes occupy ~15% of the BM in a 20 years old and as much as 60% in 65 years old (5). Adipocytes have been reported to support survival and growth of various types of tumor cells by stimulating FAO and mitochondrial OXPHOS due to high energy fatty acid transfer (6). In fact, AML cells salvage free fatty acids released by surrounding stromal adipocytes, which are then used as substrates to generate energy (7, 8).

A recent study demonstrated that BM-resident adipocytes markedly impaired the antileukemia efficacy of various chemotherapeutic agents, and that the rate of relapse after chemotherapy was higher in obese mice compared to their normal-weight counterparts (6, 9, 10). Phenotypic switching of ALM cells between the drug-sensitive and -resistant state is thought to be controlled by growth factor signaling, epigenetic regulation (11), and metabolic reprogramming (12). Furthermore, BM-resident leukemic stem cells (LSCs) exhibit a pro-inflammatory phenotype, express fatty acid transporter CD36, and induce lipolysis in BM adipocytes to fuel FAO in leukemic cells (13).

Although reports are increasing regarding the roles of altered energy metabolism during tumor progression, including AML tumorigenesis, studies focusing on fatty acid metabolism in this process remain relatively rare, and the molecular mechanisms associated with fatty acid metabolism and the tumorigenic properties of AML progenitors or LSCs are poorly characterized (14).

Interestingly, while it triggers various adaptive mechanisms that contribute to survival of AML cells, inhibition of FAO disrupts metabolic homeostasis, increases reactive oxygen species (ROS) production, and causes apoptosis in AML cells (1). Therefore, combinatorial regimens of chemotherapy with FAO inhibitors may have promise as a potential strategy to target BM-resident AML cells and LSCs. This review summarizes the current understanding of the role of fatty acid metabolism in AML cells in the adipocyte-rich BM microenvironment they occupy, and relationships between fatty acid metabolism and LSCs focusing on the potential molecular mechanisms through which AML cells acquire stemness and therapy resistance. Further, we discuss potential combinatorial regimens utilizing FAO inhibitors, which are anticipated to target the metabolic vulnerabilities of BM-resident, chemoresistant leukemia cells and LSCs.

## The BM Microenvironment Reprograms Energy Metabolism in AML Cells by Supplying Them Abundant Fatty Acids

Most tumor cells are reprogrammed to increase glucose uptake for use in glycolysis, but they generally reduce the proportion of glucose converted to acetyl-CoA that is oxidized in the Krebs cycle (1, 2), and become more dependent on FAO to drive this process (9). FAO produces over twice as much ATP per mole compared to the oxidation of glucose (15), contributes substantially to these processes under metabolic stress in cancer cells (16). Additionally, the pentose phosphate pathway utilizes glucose for the generation of NADPH that is used in the maintenance of cytosolic redox balance (17). Conversely, FAO and the Krebs cycle are an essential source of mitochondrial NADH and FADH_2_ that are oxidized in the electron transport chain to produce ATP (18).

Fatty acids are generally obtained from the extracellular microenvironment through lipolysis of stored triglycerides (19), and can act as a ligand for the peroxisome proliferator-activated receptor γ (PPARγ) located in the nucleus (20). Lipolysis of adipocytes is induced by activation of β-adrenergic receptor (21) and G protein–coupled cascade that stimulate lipolytic enzymes such as hormone-sensitive lipase (HSL) (22) and perilipinA (23). It has been reported that in the presence of ovarian cancer cells adipocytes increased release of free fatty acid with upregulation of HSL phosphorylation and Perilipin gene expression (19). Furthermore, AML blasts has been shown to induce phosphorylation of HSL and consequent activation of lipolysis in BM adipocytes (24).

BM adipocyte-supplied fatty acids are internalized via the leukemia cell scavenger receptor CD36 and transferred to the nucleus by an intracellular lipid chaperone fatty acid-binding protein 4 (FABP4) followed by ligation of PPARγ. Activated PPARγ then induces downstream target genes, including *CD36, FABP4*, and antiapoptotic *BCL2* (25). In fact, several reports have been published about the functional relationship between adipocytes and vicinal tumor cells, which is apparently mediated by FABP4-dependent mechanisms (19, 26).

In mitochondria, fatty acids that are consumed in FAO are imported in to the matrix by uncoupling protein 2 (UCP2), which can cause mitochondrial inner membrane uncoupling with the proton motive force dissipated as heat (9). Mitochondrial uncoupling in leukemia cells shifts metabolism toward FAO that negatively regulates Bak-dependent mitochondrial permeability transition (9). In leukemia cells Bcl-2 has an antioxidant function as a safeguard of mitochondrial integrity (27) by facilitating glutathione import to the mitochondrial matrix (28) or by directly reducing ROS generation (29), which results in the protection from mitochondrial uncoupling-induced apoptosis (30). Notably, it has been shown that Bcl-2 overexpression promotes low ROS-producing quiescent leukemia stem cells (31). Therefore, the combination of FAO inhibition with Bcl-2 inhibitors may improve the susceptibility of AML cells (9).

BM adipocytes also increase adiponectin receptor expression as well as its downstream target stress response kinase, AMP-activated protein kinase (AMPK) (25), which is a key modulator of energy metabolism and is activated under conditions of ATP depletion. AMPK exerts long-term metabolic control, including upregulation of fatty acid uptake, FAO, as well as autophagy regulation (32, 33). BM-adipocytes, a major source of serum adiponectin that increases during caloric restriction as well as during cancer therapy (34), have been shown to contribute to chemotherapy resistance via the secretion of adipokines as well as AMPK-dependent autophagy activation in myeloma cells (35, 36). Adiponectin-induced extracellular Ca^2+^ influx via AdipoR1is necessary for the activation of AMPK (37). BM adipocytes also activate a cancer-associated transcription factor MYC and induce an antiapoptotic chaperone heat shock protein (HSP) response in AML cells. MYC is known to stimulate the uptake of catabolites, such as fatty acids (38). HSPs that bind to denatured and unfolded proteins and promote protein refolding or degradation are positively regulated by AMPK (33), supporting AML cell survival. Thus, leukemic cells often utilize fatty acids under metabolically stressed conditions, and the NADH and FADH_2_ that are generated support ATP production, redox homeostasis, biosynthesis, as well as cell survival and proliferation.

## FAO in LSCs

LSCs are a subpopulation of AML cells in the BM microenvironment that become resistant to drugs by entering a quiescent state, which is induced by growth factor signaling, epigenetic regulation, and altered metabolism (11, 39). FAO also participates in the pathophysiological interactions between LSCs and BM stroma, which are associated with the dynamic metabolic and phenotypic reprogramming of the LSCs (40). Interaction between leukemic cells and stromal adipocytes creates a disease-specific microenvironment supporting the metabolic demands and survival of the LSC subpopulation expressing the fatty acid transporter CD36 (13). These LSCs have been shown to induce lipolysis in adipocytes, which drive FAO in LSCs and facilitates their survival (13, 24). Therefore, CD36 has an attracted attention as a new target of LSC. Sulfo-N-succinimidyl oleate (SSO) binds to CD36 and effectively blocks CD36-mediated fatty acid uptake into cardiomyocytes (41, 42), which is, however, chemically instable (41). Recently, a CD36 neutralizing antibody was shown to impair metastasis of human melanoma and breast cancer cells (43).

BM-resident LSCs exposed to adipocytes also exhibit a pro-inflammatory phenotype inducing lipolysis in vicinal adipocytes that further fuels FAO in the leukemic cells (13).

Adipocytes support survival and growth of various types of tumor cells including prostate and breast cancers by stimulating mitochondrial metabolism in tumor cells due to high energy lipid transfer (44, 45). Nieman et al. (19) has shown that co-culture of primary human omental adipocytes and ovarian cancer cells promoted lipolysis in the adipocytes and β-oxidation, invasion, and migration in the transformed cells. These activities has been mediated by adipokines including interleukin-8 (IL-8) along with upregulation of a lipid chaperone FABP4 both in omental metastases ovarian tumors. FABP4 level is also increased in AML cells cultured with BM adipocytes (25), and knockdown of a lipid chaperone FABP4 prolonged survival of a Hoxa9/Meis1-driven murine leukemia model (24). These findings suggest that FABP4 has a key role in cancer cells survival.

Recently, Jones et al. demonstrated enhanced amino acid uptake and catabolism in LSCs, and that survival of these cells isolated *de novo* from AML patients were dependent on amino acid metabolism for oxidative phosphorylation (46). On the other hand, LSCs obtained from relapsed AML patients have been shown to acquire a compensatory ability to overcome the loss of amino acid metabolism by increasing FAO (46), suggesting that the eradication of chemoresistant LSCs could be achieved by targeting LSC metabolic vulnerabilities such as FAO dependency.

## Therapeutic Options Based on Combinatorial Regimes With FAO Inhibitors

As mentioned previously, AML cells are dependent on FAO in the BM microenvironment (9). Thus, blocking FAO as a potential strategy for AML therapy. FAO inhibition could disrupt metabolic homeostasis, increase ROS, and induce the integrated stress response mediator ATF4 in AML cells causing apoptosis (47). Several studies have shown anti-AML effects of carnitine O-palmitoyltransferase 1 (CPT1) inhibition, which is a key rate-limiting enzyme in FAO (9, 47–50). CPT1 controls FAO initially by conjugating fatty acids with carnitine for translocation into the mitochondrial matrix. There are three known isoforms of CPT1, CPT1A, CPT1B, and CPT1C (51). The expression of CPT1A is reportedly regulated by PPARs and the PPARγ coactivator (PGC-1) (52), which is associated with histone deacetylase activity and enhanced tumorigenesis of breast cancer cells (51). CPT1A knockdown down-regulated mTOR signaling and increased apoptosis, and the pharmacological CPT1A inhibitor, etomoxir, sensitized leukemic cells to the chemotherapeutic drug cytarabine (AraC) (9). Although etomoxir has been frequently utilized to blockade free fatty acid entering mitochondria via CPT1, an off-target effect of etomoxir such as inhibiting complex I of the electron transport chain has been reported (52). ST1326, a novel CPT1A inhibitor, induced dose- and time-dependent cell growth arrest, mitochondrial damage, and apoptosis in primary acute lymphoid leukemia (ALL), chronic lymphoid leukemia (CLL) and particularly in AML cells (50). The association between STAT3-induced CPT1B and chemoresistance in breast cancer cells (53) as well as dysregulated CPT1B expression in bladder cancer cells (54) has also been reported.

CPT1C is regulated by AMPK causing tumor growth during metabolic stress in various cancer cell types, and CPT1C down-regulation enhanced sensitivity to rapamycin in cancer cells (55). Although etomoxir is no longer used clinically due to adverse side effects (56), other CPT1 inhibitors, including perhexiline (57), have demonstrated the ability to sensitize breast cancer cells to paclitaxel (53). A novel FAO inhibitor derived from the avocado fruit, avocatin B, decreased NADPH that is fueled by FAO through acetyl-CoA and NADH production, and caused ROS-dependent AML cell death (58, 59). The fatty acid synthase inhibitor orlistat has been shown to exhibit similar inhibitory effects on leukemia cell growth via apoptosis induction (1).

FAO inhibition can trigger reciprocal activation of bypass metabolic pathways that contribute to AML survival in the BM. This indicates possible limited efficacy of these so-called “metabolic” inhibitors when used as single agents. For example, co-culture with BM-derived adipocytes decreased the anti-leukemia effects of avocatin B along with increased glycolysis, and glucose and free fatty acid uptake in AML cells (47). Compensatory glycolysis provided continued supply of ATP to AML cells which induced pronounced lactate production. These results are consistent with the finding of increased expression of FABP4, CD36, and free fatty acid uptake in FAO-deficient Abcb11-knockout (KO) mice (60). These observations indicate that FAO inhibition triggers various adaptive mechanisms to enhance survival of AML in the BM microenvironment.

**Figure 2:**
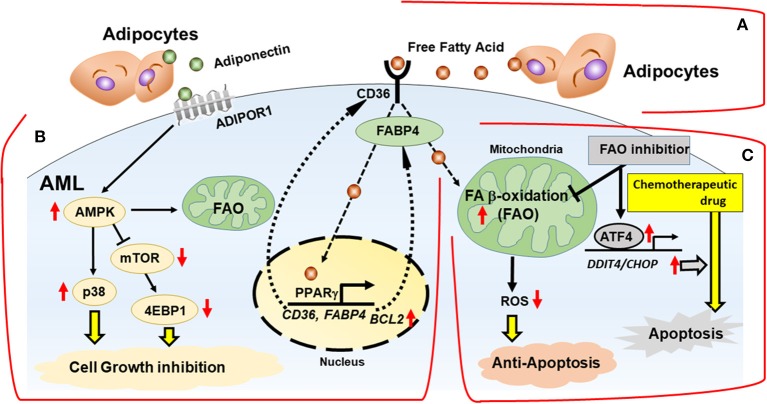
Interactions with adipocytes promotes fatty acid metabolism in AML. BM adipocytes prevent cell death of AML via FAO stimulation, with activation of AMPK and HSP chaperone proteins and modulation of transcription factors *in vitro*. **(A)** Fatty acids are obtained from the extracellular microenvironment through lipolysis of stored triglycerides in adipocytes. **(B)** BM adipocytes induce upregulation of PPARγ, CD36, and FABP4 gene transcription, which stimulates fatty acid endocytosis. The networks of transcriptional regulation and fatty acid metabolism support AML cells in a quiescent state associated with activation of AMPK, p38 with autophagy induction, upregulation of HSP anti-apoptotic chaperone proteins and chemoresistance. **(C)** In mitochondria, fatty acids are consumed for FAO, resulting in decrease of mitochondrial ROS formation and intracellular oxidative stress. FAO inhibition induces the integrated stress response, which stimulates transcriptional activation of ATF4 and facilitates apoptosis induction by chemotherapeutic drug. FABP4, fatty acid binding protein 4; AMPK, AMP-activated protein kinase; p38, p38 mitogen-activated protein kinase; ADIPOR1, adiponectin receptor 1; ATF4, activating transcription factor 4.

While FAO inhibition alone can trigger metabolic adaptation, it has been shown that FAO inhibitors are highly synergistic with conventional anti-tumor therapeutic agents like paclitaxel (53). Farge et al. (61) showed AraC-resistant AML cells displayed increased FAO and OXPHOS, and that the FAO inhibitor etomoxir induced an energy shift from high to low OXPHOS, which resulted in sensitization of these cells to AraC. Similarly, FAO inhibition by avocatin B combined with AraC caused highly synergistic effects by increasing ROS production and apoptosis in AML cells co-cultured with BM adipocytes (47). Endoplasmic reticulum (ER) stress-induced activating transcription factor 4 (ATF4) activation by avocatin B contributed to apoptosis induction by the combination treatment with AraC (47) (Figure 2). These findings suggest increased dependence on FAO in AML cells treated with AraC, which could be responsible, at least in part, for the observed synergistic apoptotic effect.

Recent reports provided promising results for simultaneous treatment with FAO inhibitors and various anti-leukemic agents. For example, in childhood acute lymphoblastic leukemia cells, L-asparaginase (ASNase), a key therapeutic target, hydrolyzed plasma asparagine and glutamine to disrupt metabolic homeostasis. ASNase also induces a compensatory increase of FAO and cellular respiration to overcome metabolic stress, and the inhibition of FAO by etomoxir markedly increased the sensitivity of acute lymphocytic leukemia (ALL) cells to ASNase (62).

Shinohara et al. reported that inactivation of BCR-ABL by the tyrosine kinase inhibitor imatinib strongly suppressed glycolysis and a compensatory increase of FAO by up-regulating CPT1C in Ph-positive ALL and chronic myeloid leukemia (CML) cells. The fatty-acid derivative and FAO inhibitor AIC-47 reversed the TKI-induced upregulation of FAO and exhibited synergic cytotoxicity with imatinib (63, 64). In chronic lymphocytic leukemia (CLL), high-dose glucocorticoids (GCs), induced activation of PPARα and downstream FAO that confer resistance. Tung et al. demonstrated that PPARα and FAO enzyme inhibitors increased the cytotoxicity of dexamethasone in CLL cells *in vitro* and *in vivo* (65). These findings highlight the potential of combination regimens with FAO inhibitors that target metabolic vulnerabilities of BM-resident chemoresistant leukemia cells.

## Conclusions and Future Directions

Here we have described recent evidence for the role of fatty acid metabolism and FAO in AML survival in the adipocyte-rich BM microenvironment. Metabolic alterations in leukemia cells have drawn increasing attention as an important therapeutic objective, and encouraging results with reagents that targeting fatty acid metabolism have been achieved in preclinical leukemia models. Leukemic cells may be critically dependent on certain metabolic pathways as their molecular heterogeneity suggest. Hence, the metabolism changes in leukemia cells vs. their normal hematopoietic counterparts can be used to generate a therapeutic window of selective sensitivity for the tumor cells. Nevertheless, tumor cells can adapt quickly to nutrient deprivation via activation of multiple metabolic bypass processes with support by their microenvironment. In this manner, FAO inhibition triggers various adaptive mechanisms that enhance AML survival in the BM, suggesting the inadequate usefulness of FAO inhibitors when used as alone (47). Alternatively, FAO inhibition combined with the conventional chemotherapy or targeted therapeutics has the potential to synergistically eradicate BM-resident, chemoresistant AML cells.

In conclusion, recent studies have highlighted the importance of understanding AML cell metabolism in the BM microenvironment, and emphasize the potential of drug combinatorial strategies aiming at metabolic vulnerabilities of resistant AML cells and LSCs. The metabolic adaptation of AML cells and the variation of fatty acid metabolism signify an attractive therapeutic target for use against hematological malignancies. Emphasis should be placed on ROS as the executioners of cytotoxicity by metabolism-targeting therapeutics, and predictive biomarkers of response to these drugs should be explored, since both are critical to improving clinical outcomes. More comprehensive mechanistic findings from future studies may reveal that therapeutic targeting of fatty acid metabolism and FAO, especially in elderly patients with increased adiposity in the BM microenvironment, will hopefully increase the efficacy of related chemotherapeutic agents as well as reduce their toxicity.

## Author Contributions

All authors have contributed to the preparation of the manuscript and reviewed/approved it in its final form.

### Conflict of Interest

The authors declare that the research was conducted in the absence of any commercial or financial relationships that could be construed as a potential conflict of interest.

## References

[1] Jones RG Thompson CB. Tumor suppressors and cell metabolism: a recipe for cancer growth. Genes Dev. (2009) 23:537–48. 10.1101/gad.1756509 19270154 PMC2763495

[2] Vander Heiden MG Cantley LC Thompson CB. Understanding the Warburg effect: the metabolic requirements of cell proliferation. Science. (2009) 324:1029–33. 10.1126/science.1160809 19460998 PMC2849637

[3] Pietrocola F Galluzzi L Bravo-San Pedro JM Madeo F Kroemer G. Acetyl coenzyme A: a central metabolite and second messenger. Cell Metab. (2015) 21:805–21. 10.1016/j.cmet.2015.05.014 26039447

[4] Hassan M Abedi-Valugerdi M. Hematologic malignancies in elderly patients. Haematologica. (2014) 99:1124–7. 10.3324/haematol.2014.107557 24986872 PMC4077070

[5] Justesen J Stenderup K Ebbesen EN Mosekilde L Steiniche T Kassem M. Adipocyte tissue volume in bone marrow is increased with aging and in patients with osteoporosis. Biogerontology. (2001) 2:165–71. 10.1023/A:1011513223894 11708718

[6] Behan JW Yun JP Proektor MP Ehsanipour EA Arutyunyan A Moses AS. Adipocytes impair leukemia treatment in mice. Cancer Res. (2009) 69:7867–74. 10.1158/0008-5472.CAN-09-0800 19773440 PMC2756308

[7] Röhrig F Schulze A. The multifaceted roles of fatty acid synthesis in cancer. Nat Rev Cancer. (2016) 16:732–49. 10.1038/nrc.2016.89 27658529

[8] Beloribi-Djefaflia S Vasseur S Guillaumond F. Lipid metabolic reprogramming in cancer cells. Oncogenesis. (2016) 5:e189. 10.1038/oncsis.2015.49 26807644 PMC4728678

[9] Samudio I Harmancey R Fiegl M Kantarjian H Konopleva M Korchin B. Pharmacologic inhibition of fatty acid oxidation sensitizes human leukemia cells to apoptosis induction. J Clin Invest. (2010) 120:142–56. 10.1172/JCI38942 20038799 PMC2799198

[10] Fiegl M Samudio I Clise-Dwyer K Burks JK Mnjoyan Z Andreeff M. CXCR4 expression and biologic activity in acute myeloid leukemia are dependent on oxygen partial pressure. Blood. (2009) 113:1504–12. 10.1182/blood-2008-06-161539 18957686 PMC2644078

[11] Sharma SV Lee DY Li B Quinlan MP Takahashi F Maheswaran S. A chromatin-mediated reversible drug-tolerant state in cancer cell subpopulations. Cell. (2010) 141:69–80. 10.1016/j.cell.2010.02.027 20371346 PMC2851638

[12] Hirsch HA Iliopoulos D Joshi A Zhang Y Jaeger SA Bulyk M. A transcriptional signature and common gene networks link cancer with lipid metabolism and diverse human diseases. Cancer Cell. (2010) 17:348–61. 10.1016/j.ccr.2010.01.022 20385360 PMC2854678

[13] Ye H Adane B Khan N Sullivan T Minhajuddin M Gasparetto M. Leukemic stem cells evade chemotherapy by metabolic adaptation to an adipose tissue niche. Cell Stem Cell. (2016) 19:23–37. 10.1016/j.stem.2016.06.001 27374788 PMC4938766

[14] Lobo NA Shimono Y Qian D Clarke MF. The biology of cancer stem cells. Annu Rev Cell Dev Biol. (2007) 23:675–99. 10.1146/annurev.cellbio.22.010305.104154 17645413

[15] Boroughs LK DeBerardinis RJ. Metabolic pathways promoting cancer cell survival and growth. Nat Cell Biol. (2015) 17:351–9. 10.1038/ncb3124 25774832 PMC4939711

[16] Jeon SM Chandel NS Hay N. AMPK regulates NADPH homeostasis to promote tumour cell survival during energy stress. Nature. (2012) 485:661–5. 10.1038/nature11066 22660331 PMC3607316

[17] Kruiswijk F Labuschagne CF Vousden KH. p53 in survival, death and metabolic health: a lifeguard with a licence to kill. Nat Rev Mol Cell Biol. (2015) 16:393–405. 10.1038/nrm4007 26122615

[18] Carracedo A Cantley LC Pandolfi PP. Cancer metabolism: fatty acid oxidation in the limelight. Nat Rev Cancer. (2013) 13:227–32. 10.1038/nrc3483 23446547 PMC3766957

[19] Nieman KM Kenny HA Penicka CV Ladanyi A Buell-Gutbrod R Zillhardt MR. Adipocytes promote ovarian cancer metastasis and provide energy for rapid tumor growth. Nat Med. (2011) 17:1498–503. 10.1038/nm.2492 22037646 PMC4157349

[20] Itoh T Fairall L Amin K Inaba Y Szanto A Balint BL. Structural basis for the activation of PPARgamma by oxidized fatty acids. Nat Struct Mol Biol. (2008) 15:924–31. 10.1038/nsmb.1474 19172745 PMC2939985

[21] Carmen GY Victor SM. Signalling mechanisms regulating lipolysis. Cell Signal. (2006) 18:401–8. 10.1016/j.cellsig.2005.08.009 16182514

[22] Sengenes C Bouloumie A Hauner H Berlan M Busse R Lafontan M. Involvement of a cGMP-dependent pathway in the natriuretic peptide-mediated hormone-sensitive lipase phosphorylation in human adipocytes. J Biol Chem. (2003) 278:48617–26. 10.1074/jbc.M303713200 12970365

[23] Brasaemle DL Subramanian V Garcia A Marcinkiewicz A Rothenberg A. Perilipin A and the control of triacylglycerol metabolism. Mol Cell Biochem. (2009) 326:15–21. 10.1007/s11010-008-9998-8 19116774

[24] Shafat MS Oellerich T Mohr S Robinson SD Edwards DR Marlein CR. Leukemic blasts program bone marrow adipocytes to generate a protumoral microenvironment. Blood. (2017) 129:1320–32. 10.1182/blood-2016-08-734798 28049638

[25] Tabe Y Yamamoto S Saitoh K Sekihara K Monma N Ikeo K. Bone marrow adipocytes facilitate fatty acid oxidation activating AMPK and a transcriptional network supporting survival of acute monocytic leukemia cells. Cancer Res. (2017) 77:1453–64. 10.1158/0008-5472.CAN-16-1645 28108519 PMC5354955

[26] Herroon MK Rajagurubandara E Hardaway AL Powell K Turchick A Feldmann D. Bone marrow adipocytes promote tumor growth in bone via FABP4-dependent mechanisms. Oncotarget. (2013) 4:2108–23. 10.18632/oncotarget.1482 24240026 PMC3875773

[27] Velez J Hail N Jr Konopleva M Zeng Z Kojima K Samudio I. Mitochondrial uncoupling and the reprograming of intermediary metabolism in leukemia cells. Front Oncol. (2013) 3:67. 10.3389/fonc.2013.00067 23565503 PMC3613776

[28] Wilkins HM Marquardt K Lash LH Linseman DA. Bcl-2 is a novel interacting partner for the 2-oxoglutarate carrier and a key regulator of mitochondrial glutathione. Free Radic Biol Med. (2012) 52:410–9. 10.1016/j.freeradbiomed.2011.10.495 22115789 PMC3253244

[29] Low IC Chen ZX Pervaiz S. Bcl-2 modulates resveratrol-induced ROS production by regulating mitochondrial respiration in tumor cells. Antioxid Redox Signal. (2010) 13:807–19. 10.1089/ars.2009.3050 20367277

[30] Armstrong JS Steinauer KK French J Killoran PL Walleczek J Kochanski J. Bcl-2 inhibits apoptosis induced by mitochondrial uncoupling but does not prevent mitochondrial transmembrane depolarization. Exp Cell Res. (2001) 262:170–9. 10.1006/excr.2000.5091 11139341

[31] Lagadinou ED Sach A Callahan K Rossi RM Neering SJ Minhajuddin M. BCL-2 inhibition targets oxidative phosphorylation and selectively eradicates quiescent human leukemia stem cells. Cell Stem Cell. (2013) 12:329–41. 10.1016/j.stem.2012.12.013 23333149 PMC3595363

[32] Yamauchi T Kamon J Minokoshi Y Ito Y Waki H Uchida S. Adiponectin stimulates glucose utilization and fatty-acid oxidation by activating AMP-activated protein kinase. Nat Med. (2002) 8:1288–95. 10.1038/nm788 12368907

[33] Alers S Löffler AS Wesselborg S Stork B. Role of AMPK-mTOR-Ulk1/2 in the regulation of autophagy: cross talk, shortcuts, and feedbacks. Mol Cell Biol. (2012) 32:2–11. 10.1128/MCB.06159-11 22025673 PMC3255710

[34] Cawthorn WP Scheller EL Learman BS Parlee SD Simon BR Mori H. Bone marrow adipose tissue is an endocrine organ that contributes to increased circulating adiponectin during caloric restriction. Cell Metab. (2014) 20:368–75. 10.1016/j.cmet.2014.06.003 24998914 PMC4126847

[35] Medina EA Oberheu K Polusani SR Ortega V Velagaleti GV Oyajobi BO. PKA/AMPK signaling in relation to adiponectin's antiproliferative effect on multiple myeloma cells. Leukemia. (2014) 28:2080–9. 10.1038/leu.2014.112 24646889

[36] Gwinn DM Shackelford DB Egan DF Mihaylova MM Mery A Vasquez DS. AMPK phosphorylation of raptor mediates a metabolic checkpoint. Mol Cell. (2008) 30:214–26. 10.1016/j.molcel.2008.03.003 18439900 PMC2674027

[37] Iwabu M Yamauchi T Okada-Iwabu M Sato K Nakagawa T Funata M. Adiponectin and AdipoR1 regulate PGC-1alpha and mitochondria by Ca(2+) and AMPK/SIRT1. Nature. (2010) 464:1313–9. 10.1038/nature08991 20357764

[38] Stine ZE Walton ZE Altman BJ Hsieh AL Dang CV. MYC, metabolism, and cancer. Cancer Discov. (2015) 5:1024–39. 10.1158/2159-8290.CD-15-0507 26382145 PMC4592441

[39] Sancho P Barneda D Heeschen C. Hallmarks of cancer stem cell metabolism. Br J Cancer. (2016) 114:1305–12. 10.1038/bjc.2016.152 27219018 PMC4984474

[40] Chaffer CL Brueckmann I Scheel C Kaestli AJ Wiggins PA Rodrigues LO. Normal and neoplastic non-stem cells can spontaneously convert to a stem-like state. Proc Natl Acad Sci USA. (2011) 108:7950–5. 10.1073/pnas.1102454108 21498687 PMC3093533

[41] Coort SL Willems J Coumans WA van der Vusse GJ Bonen A Glatz JF. Sulfo-N-succinimidyl esters of long chain fatty acids specifically inhibit fatty acid translocase (FAT/CD36)-mediated cellular fatty acid uptake. Mol Cell Biochem. (2002) 239:213–9. 10.1023/A:1020539932353 12479588

[42] Greenwalt DE Scheck SH Rhinehart-Jones T. Heart CD36 expression is increased in murine models of diabetes and in mice fed a high fat diet. J Clin Invest. (1995) 96:1382–8. 10.1172/JCI118173 7544802 PMC185760

[43] Pascual G Avgustinova A Mejetta S Martin M Castellanos A Attolini CS. Targeting metastasis-initiating cells through the fatty acid receptor CD36. Nature. (2017) 541:41–5. 10.1038/nature20791 27974793

[44] Tokuda Y Satoh Y Fujiyama C Toda S Sugihara H Masaki Z. Prostate cancer cell growth is modulated by adipocyte-cancer cell interaction. BJU Int. (2003) 91:716–20. 10.1046/j.1464-410X.2003.04218.x 12699491

[45] Dirat B Bochet L Dabek M Daviaud D Dauvillier S Majed B. Cancer-associated adipocytes exhibit an activated phenotype and contribute to breast cancer invasion. Cancer Res. (2011) 71:2455–65. 10.1158/0008-5472.CAN-10-3323 21459803

[46] Jones CL Stevens BM D'Alessandro A Reisz JA Culp-Hill R Nemkov T. Inhibition of amino acid metabolism selectively targets human leukemia stem cells. Cancer Cell. (2019) 35:333–5. 10.1016/j.ccell.2019.01.013 30753831 PMC6389327

[47] Tabe Y Saitoh K Yang H Sekihara K Yamatani K Ruvolo V. Inhibition of FAO in AML co-cultured with BM adipocytes: mechanisms of survival and chemosensitization to cytarabine. Sci Rep. (2018) 8:16837. 10.1038/s41598-018-35198-6 30442990 PMC6237992

[48] Qu Q Zeng F Liu X Wang QJ Deng F. Fatty acid oxidation and carnitine palmitoyltransferase I: emerging therapeutic targets in cancer. Cell Death Dis. (2016) 7:e2226. 10.1038/cddis.2016.132 27195673 PMC4917665

[49] Pike LS Smift AL Croteau NJ Ferrick DA Wu M. Inhibition of fatty acid oxidation by etomoxir impairs NADPH production and increases reactive oxygen species resulting in ATP depletion and cell death in human glioblastoma cells. Biochim Biophys Acta. (2011) 1807:726–34. 10.1016/j.bbabio.2010.10.022 21692241

[50] Ricciardi MR Mirabilii S Allegretti M Licchetta R Calarco A Torrisi MR. Targeting the leukemia cell metabolism by the CPT1a inhibition: functional preclinical effects in leukemias. Blood. (2015) 126:1925–9. 10.1182/blood-2014-12-617498 26276667

[51] Schreurs M Kuipers F van der Leij FR. Regulatory enzymes of mitochondrial beta-oxidation as targets for treatment of the metabolic syndrome. Obes Rev. (2010) 11:380–8. 10.1111/j.1467-789X.2009.00642.x 19694967

[52] Yao CH Liu GY Wang R Moon SH Gross RW Patti GJ. Identifying off-target effects of etomoxir reveals that carnitine palmitoyltransferase I is essential for cancer cell proliferation independent of beta-oxidation. PLoS Biol. (2018) 16:e2003782. 10.1371/journal.pbio.2003782 29596410 PMC5892939

[53] Wang T Fahrmann JF Lee H Li YJ Tripathi SC Yue C JAK/STAT3-regulated fatty acid β-oxidation is critical for breast cancer stem cell self-renewal and chemoresistance. Cell Metab. (2018) 27:1357 10.1016/j.cmet.2018.04.018 PMC6116734 29874570

[54] Kim WT Yun SJ Yan C Jeong P Kim YH Lee IS. Metabolic pathway signatures associated with urinary metabolite biomarkers differentiate bladder cancer patients from healthy controls. Yonsei Med J. (2016) 57:865–71. 10.3349/ymj.2016.57.4.865 27189278 PMC4951461

[55] Zaugg K Yao Y Reilly PT Kannan K Kiarash R Mason J. Carnitine palmitoyltransferase 1C promotes cell survival and tumor growth under conditions of metabolic stress. Genes Dev. (2011) 25:1041–51. 10.1101/gad.1987211 21576264 PMC3093120

[56] Holubarsch CJ Rohrbach M Karrasch M Boehm E Polonski L Ponikowski P. A double-blind randomized multicentre clinical trial to evaluate the efficacy and safety of two doses of etomoxir in comparison with placebo in patients with moderate congestive heart failure: the ERGO (etomoxir for the recovery of glucose oxidation) study. Clin Sci. (2007) 113:205–12. 10.1042/CS20060307 17319797

[57] Yin X Dwyer J Langley SR Mayr U Xing Q Drozdov I. Effects of perhexiline-induced fuel switch on the cardiac proteome and metabolome. J Mol Cell Cardiol. (2013) 55:27–30. 10.1016/j.yjmcc.2012.12.014 23277191 PMC3573230

[58] Lee EA Angka L Rota SG Hanlon T Mitchell A Hurren R. Targeting mitochondria with avocatin B induces selective leukemia cell death. Cancer Res. (2015) 75:2478–88. 10.1158/0008-5472.CAN-14-2676 26077472

[59] Tcheng M Samudio I Lee EA Minden MD Spagnuolo PA. The mitochondria target drug avocatin B synergizes with induction chemotherapeutics to induce leukemia cell death. Leuk Lymphoma. (2017) 58:986–8. 10.1080/10428194.2016.1218005 27558298

[60] Zhang Y Li F Patterson AD Wang Y Krausz KW Neale G. Abcb11 deficiency induces cholestasis coupled to impaired β-fatty acid oxidation in mice. J Biol Chem. (2012) 287:24784–94. 10.1074/jbc.M111.329318 22619174 PMC3397905

[61] Farge T Saland E de Toni F Aroua N Hosseini M Perry R. Chemotherapy resistant human acute myeloid leukemia cells are not enriched for leukemic stem cells but require oxidative metabolism. Cancer Discov. (2017) 7:716–35. 10.1158/2159-8290.CD-16-0441 28416471 PMC5501738

[62] Hermanova I Arruabarrena-Aristorena A Valis K Nuskova H Alberich-Jorda M Fiser K. Pharmacological inhibition of fatty-acid oxidation synergistically enhances the effect of l-asparaginase in childhood ALL cells. Leukemia. (2016) 30:209–18. 10.1038/leu.2015.213 26239197

[63] Shinohara H Kumazaki M Minami Y Ito Y Sugito N Kuranaga Y. Perturbation of energy metabolism by fatty-acid derivative AIC-47 and imatinib in BCR-ABL-harboring leukemic cells. Cancer Lett. (2016) 371:1–11. 10.1016/j.canlet.2015.11.020 26607903

[64] Shinohara H Sugito N Kuranaga Y Heishima K Minami Y Naoe T. Potent antiproliferative effect of fatty-acid derivative AIC-47 on leukemic mice harboring BCR-ABL mutation. Cancer Sci. (2019) 110:751–60. 10.1111/cas.13913 30548479 PMC6361563

[65] Tung S Shi Y Wong K Zhu F Gorczynski R Laister RC. PPARα and fatty acid oxidation mediate glucocorticoid resistance in chronic lymphocytic leukemia. Blood. (2013) 122:969–80. 10.1182/blood-2013-03-489468 23814018

